# Variations in health literacy and influential factors affecting the categories of social support among rural patients with diabetes mellitus

**DOI:** 10.3389/fpubh.2024.1373591

**Published:** 2024-10-30

**Authors:** Xin Zhang, Yan-Ping Zhang, Lin Zeng, Xiang Li, Jia-Xia Han, Gui-Fen Fu, Chao-Qun Bai, Xiao-Xue Lei

**Affiliations:** ^1^Department of Geriatric Endocrinology and Metabolism, Guangxi Academy of Medical Sciences and the People's Hospital of Guangxi Zhuang Autonomous Region, Nanning, China; ^2^Department of Otorhinolaryngology, The People's Hospital of Guangxi Zhuang Autonomous Region, Guangxi Academy of Medical Sciences, Nanning, China; ^3^Department of Endocrinology and Metabolism, Guangxi Academy of Medical Sciences and the People's Hospital of Guangxi Zhuang Autonomous Region, Nanning, China; ^4^Department of Nursing, Guangxi Academy of Medical Sciences and the People's Hospital of Guangxi Zhuang Autonomous Region, Nanning, China

**Keywords:** health literacy, latent profile analysis (LPA), social support, diabetes mellitus (DM), rural areas of China

## Abstract

**Objective:**

The aim of this study is to explore the inherent classification of social support among individuals residing in rural areas of China. Additionally, we aim to examine the attributes and variations in health literacy scores among patients with diabetes mellitus (DM) within diverse social support categories.

**Design:**

Cross-sectional study.

**Methods:**

Employing the multi-stage stratified sampling technique, we enrolled 2,178 patients diagnosed with DM residing in the rural regions of Guangxi Province. We utilized the General Information Questionnaire, Social Support Rating Scale (SSRS), and Functional, Communicative and Critical Health Literacy Instrument.

**Results:**

The rural patients with DM were categorized into four distinct groups based on the types and levels of their underlying social support. These groups included a low-level social support utilization group (43%), a low-level objective social support group (17%), a moderate-level social support group (20%), and a high-level social support and high-level utilization group (20%). Statistical analysis revealed significant differences among the four groups in terms of age, disease duration, and blood sugar control level (*p* < 0.05). Furthermore, health literacy scores and scores across various dimensions for rural patients with DM demonstrated variability in accordance with latent profiles of social support, with statistically significant differences observed (*p* < 0.05). A positive correlation was identified between the level of social support and all dimensions of health literacy among rural patients with DM (*p* < 0.05).

**Conclusion:**

The social support available to individuals with DM in rural settings can be categorized into four distinct types, and its manifestation is influenced by demographic factors. The health literacy of rural patients with DM is intricately linked to the extent of social support they receive. For enhanced outcomes, interventions targeted at enhancing health literacy and quality of life among rural patients with DM should be tailored to address the heterogeneity observed in latent profiles of social support.

## 1 Introduction

Diabetes mellitus (DM) is a persistent metabolic disorder characterized by elevated blood glucose levels, influenced by genetic, environmental, and other factors (1). Given its widespread prevalence and the associated rates of disability and mortality, DM poses a significant global public health threat (2). Surveys indicate a higher incidence of DM in rural compared to urban areas in China (3). The scarcity of grassroots health resources in these regions leads to delayed medical intervention and a lack of disease-related knowledge, contributing to increased complication rates (4).

Social support encompasses assistance that individuals receive from their social networks, including family, friends, colleagues, and organizations (5). Within the framework of latent profile analysis (LPA), research participants are systematically classified according to quantifiable inquiries, thereby elucidating distinctive characteristics and identifying influential factors across diverse groups (6). Past studies often used aggregate scores to assess social support levels, overlooking individual-level variations. Research indicates a correlation between social support and improved blood sugar control in patients with DM, where heightened support fosters positive attitudes and encourages beneficial lifestyle changes (7, 8).

Patients with DM with good health literacy can access, comprehend, and manage essential health information (9). Similarly, patients with higher social support have better health literacy and can better use the obtained health resources. There is a recognized correlation between social support and health literacy, with robust health literacy elevating social support levels (10). In this study, we focused on rural patients with DM, aiming to examining the attributes and variations in health literacy scores among patients with diabetes mellitus (DM) within diverse social support categories. Additionally, we explored influential factors affecting the categories of social support in this population. These findings may provide valuable insights for clinicians to tailor more effective blood sugar management plans and enhance health literacy levels among diverse rural patients.

## 2 Methods

### 2.1 Study participants

The multi-stage stratified sampling method was utilized in this study, and sampling was conducted from January 2022 to July 2022. In the first stage, five cities (Nanning, Guilin, Hechi, Chongzuo, and Yulin) were randomly selected from geographical regions (eastern, western, southern, northern, and central) of the Guangxi Zhuang Autonomous Region. In the second stage, three counties were randomly chosen from each of the selected cities, leading to a survey of 15 counties. A total of 2,280 patients were involved in the investigation. The inclusion criteria were: (1) Individuals fulfilled the diagnostic criteria for DM as outlined by the Chinese Diabetes Society in the 2020 edition (11); (2) Individuals aged 18 years or older; (3) Individuals whose officially registered residence and current living location fell within the surveyed regions. Participants with (1) gestational DM, (2) dementia or similar mental health conditions, or (3) in the acute phase of the disease who were unable to participate in the survey due to their condition were excluded. Informed consent was obtained from the administrators of the participating hospitals, and written informed consent was obtained from all study participants. The study protocol received approval from the Ethics Committee of the People's Hospital of Guangxi Zhuang Autonomous Region under the ethics number KT-KJT-2021-26.

### 2.2 Study tools

#### 2.2.1 General information questionnaire

We designed a General Information Questionnaire that encompassed demographic details such as age, gender, marital status, educational attainment, and average annual per capita disposable income of households [categorized based on the 2021 China National Bureau of Statistics standards for rural residents' income, set at RMB 18,931 per year (12)]. Disease-related information encompassed disease duration, glycated hemoglobin levels, use of oral diabetes medication, and history of insulin therapy, among other relevant factors.

#### 2.2.2 Social support rating scale

The Social Support Rating Scale (SSRS), developed by Xiao Shuiyuan, was utilized to gauge the extent of social support in this study (13). Comprising 3 dimensions and 10 items, the scale includes 4 items for subjective support, 3 for objective support, and 3 for the utilization of social support. Scores on the scale range from 12 to 66 points, with higher scores signifying increased social support for the study participants. The retest reliability of the scale is 0.92, and each item demonstrates internal consistency reliability ranging from 0.89 to 0.94, as assessed by Cronbach's α coefficient. These findings affirm the scale's robust reliability and validity. Widely employed in Chinese research, the SSRS serves as a well-established tool for evaluating social support levels.

#### 2.2.3 Functional, communicative, and critical health literacy instrument (FCCHLI)

The FCCHLI, initially developed by Ishikawa et al. (14) in 2008 to assess the health literacy of individuals with DM, was localized for application in China. Zhao et al. conducted reliability and effectiveness testing of the scale within the Chinese context in 2021 (15). This scale evaluates three dimensions: functional health literacy, communicative health literacy, and critical health literacy, encompassing a total of 14 items. Utilizing a 4-point Likert scale for scoring, the functional health literacy dimension is scored in reverse, with a lower score indicative of a higher level of health literacy. The total score ranges from 14 to 56 points. The Cronbach's α coefficient for the Chinese version of the FCCHLI is 0.868. A reliability analysis of the FCCHL was performed using collected and organized data, resulting in a Cronbach's α coefficient of 0.833, indicating high reliability.

### 2.3 Statistical methods

The data was processed utilizing SPSS 27.0 statistical software, encompassing statistical descriptions of general information, single-factor analysis, correlation analysis, and regression analysis. Statistical significance was established with a threshold of a *p*-value below 0.05 (*p* < 0.05). Furthermore, Latent Profile Analysis (LPA) was performed employing Mplus 8.3 software, with the scores of the 10 items from the SSRS serving as the manifest variables. Subsequently, profiles 1 through 6 were chosen for analysis. The final model fit was evaluated using the following indicators (16): (1) Information Metrics: The information metrics comprised Akaike Information Criterion (AIC), Bayesian Information Criterion (BIC), and Sample-Size Adjusted Bayesian Information Criterion (aBIC). Lower values of these indicators are indicative of superior model fit. (2) Likelihood ratio test (LRT) metrics, including the Lo-Mendell-Rubin adjusted likelihood-ratio test (LMR-A-LRT) and Bootstrap Likelihood Ratio Test (BLRT), were employed to assess disparities in model fit between the K-class model and the K-1-class model. (3) Entropy: A maximum entropy value of 1 was observed, with elevated values signifying greater precision in categorization. (4) Group Size: Typically, each group must constitute more than 5% of the overall sample size.

## 3 Results

### 3.1 General information of study participants

In this study, a total of 2,280 survey questionnaires were disseminated, with 2,178 responses received, yielding a response rate of 95.52%. The demographic composition of the respondents is delineated as follows: 1,204 identified as male and 974 as female. The distribution across age groups revealed 1,348 participants aged 60 years or older. Among the participants, 2,020 were married. Educational backgrounds varied, encompassing 256 individuals with no formal education (11.70%), 504 with primary school education (23.20%), 684 with junior high school education (31.40%).

The survey also captured economic disparities, with 822 respondents reporting an average annual per capita disposable income of RMB 18,931 or above, and 1,356 respondents with incomes below this threshold. Regarding the duration of the disease, 1,134 cases (52.1%) between 5 and 10 years, and 579 cases (26.6%) over 10 years. Additional comprehensive data are shown in Table 1.

**Table 1:** Demographic details of the study participants (***n*** **=** 2,178).

**Item**	**Group**	**Cases (percentage, %)**
Age (years)	18–44	158 (7.3)
	45–59	672 (30.8)
	60-	1,348 (61.9)
Gender	Male	1,204 (55.3)
	Female	974 (44.7)
Marital status	Married	2,020 (92.7)
	Unmarried or others	158 (7.3)
Educational level	Illiterate	256 (11.8)
	Primary school	504 (23.1)
	Junior high school	684 (31.4)
	Senior high school	507 (23.3)
	Three-year junior college	180 (8.3)
	Bachelor	47 (2.2)
Average annual per capita disposable income of households	< RMB 18,931	1,356 (62.3)
	≥RMB 18,931	822 (37.7)
Employment status	Employed	177 (8.1)
	Unemployed	2,001 (91.9)
Duration of the illness (years)	< 5	465 (21.3)
	5–10	1,134 (52.1)
	>10	579 (26.6)
Family history of DM	Yes	317 (14.6)
	No	1,861 (85.4)
Control of glycated hemoglobin (HbA1c)	< 7.0	494 (22.7)
	≥7.0	1,684 (77.3)
Status of oral diabetes medication administration	Yes	1,349 (61.9)
	No	829 (38.1)
Status of insulin administration	Yes	913 (41.9)
	No	1,265 (58.1)
Number of complications	0	607 (27.9)
	1	895 (41.1)
	2	554 (25.4)
	3	112 (5.1)
	4	10 (0.5)

### 3.2 Latent profile analysis

The social support scores of rural patients with DM were classified into six categories (Classes 1–6) and analyzed using LPA for model fitting, as detailed in Table 2. The findings revealed a consistent reduction in both AIC and BIC values with the sequential progression of class numbers. Although the 6th model retained statistical significance in terms of the LMR (P) value, its significance was diminished compared to the 5th model.

**Table 2:** Indicators for latent profiles of social support among rural patients with DM.

**Model**	***AIC***	***BIC***	***aBIC***	***ENTYOPY***	***LMR (P)***	***BLRT (P)***	**Group size (%)**
1	78,236.313	78,440.037	78,376.494	-	-	-	-
2	73,926.517	74,102.788	74,004.297	0.972	< 0.001	< 0.001	0.22/0.78
3	71,214.439	71,453.258	71,319.818	0.994	< 0.001	< 0.001	0.17/0.61/0.22
4	67,368.01	67,669.377	67,500.989	0.998	< 0.001	< 0.001	0.43/0.17/0.2/0.2
5	65,524.677	65,888.592	65,685.255	0.998	< 0.001	< 0.001	0.15/0.43/0.27/0.06/0.09
6	61,496.231	61,992.693	61,684.408	0.997	0.0048	< 0.001	0.11/0.26/0.35/0.05/0.13/0.1

AIC, Akaike Information Criterion; BIC, Bayesian Information Criterion; aBIC, Sample-Size Adjusted Bayesian Information Criterion; LRT, Likelihood ratio test; LMR-A-LRT, Lo-Mendell-Rubin adjusted likelihood-ratio test; BLRT, Bootstrap Likelihood Ratio Test.

Entropy values remained stable for the 4th and 5th models. Ultimately, after a thorough evaluation of statistical analyses and scale scores, the 4-class model was chosen as the most appropriate latent profile model. The average membership probabilities for the four latent profiles were 99.9%, affirming the rationality of the model. Social support scores for the four categories were 20.52 ± 2.96, 21.75 ± 3.54, 26.87 ± 6.35, and 44.09 ± 4.47, respectively.

The first group, characterized by significantly lower social support utilization, was named the low-level social support utilization group. The second group, with a higher total score but significantly lower objective social support, was termed the low-level objective social support group. The third group exhibited moderate total scores and scores across all dimensions, denoted as the moderate-level social support group. The fourth group displayed notably higher total scores, particularly in subjective social support, earning the designation of the high-level social support and high-level utilization group. This distinction may be attributed to the wider scoring range of the 5th question within the scale.

### 3.3 Examining the correlation between social support scores across various dimensions and health literacy in individuals diagnosed with DM residing in rural areas

The social support score for rural patients with DM was 26.7 ± 9.93 points. Subjective social support had the highest score at 10.66 ± 6.01 points, whereas social support utilization had the lowest score at 7.29 ± 1.69 points. The health literacy score of rural diabetic patients was 30.47±9.36 points, and the highest score was the functional dimension, with a score of 11.17±3.76 points. All scores are shown in Table 3. Significant positive correlations were observed between the social support score, scores in all dimensions, and health literacy. The correlation between communicative health literacy and social support is the highest, which is 0.437 (*p*<*0.01*) (Table 4).

**Table 3:** Social support and health literacy scores by dimension for rural diabetes patients.

**Item**	**Maximum value**	**Minimal value**	**Score (χ ±*s*)**
Subjective support	28	5	10.66 ± 6.01
Objective support	19	3	8.75 ± 4.39
Utilization of support	11	4	7.29 ± 1.69
Total social support	52	13	26.7 ± 9.93
Communication	19	5	10.64 ± 3.93
Functional	17	5	11.17 ± 3.76
Critical	16	4	8.65 ± 3.07
Total health literacy	48	16	30.47 ± 9.36

**Table 4:** Correlation analysis of social support and health literacy in rural diabetes patients.

**Item**	**Health literacy**	**Communication**	**Functional**	**Critical**
Social support	0.482^**^	0.437^**^	0.409^**^	0.398^**^
Subjective support	0.378^**^	0.354^**^	0.316^**^	0.312^**^
Objective support	0.378^**^	0.339^**^	0.321^**^	0.323^**^
Utilization of support	0.208^**^	0.183^**^	0.179^**^	0.180^**^

^**^p < 0.01.

### 3.4 Assessment of health literacy scores among four distinct groups of rural patients with DM

The health literacy scores for the four groups, along with scores in different dimensions, exhibited a statistically significant increase corresponding to the rise in group serial numbers, as illustrated in Table 5.

**Table 5:** Comparison of health literacy scores among rural patients with DM across different categories of social support.

	**Low-level social support utilization group (*n* = 939)**	**Low-level objective social support group (*n* = 361)**	**Moderate-level social support group (*n* = 446)**	**High-level social support and high-level utilization group (*n* = 432)**	***F***	***P***
Total score of health literacy	27.16 ± 8.54	28.49 ± 8.78	31.81 ± 8.83	37.92 ± 7.33	171.644	< 0.001
Functional health literacy	10.03 ± 3.6	10.5 ± 3.67	11.69 ± 3.65	13.66 ± 2.91	113.922	< 0.001
Communicative health literacy	9.38 ± 3.63	9.98 ± 3.68	11.11 ± 3.76	13.46 ± 3.35	132.862	< 0.001
Critical health literacy	7.75 ± 2.82	8.01 ± 3.01	9.01 ± 3.04	10.79 ± 2.57	122.151	< 0.001

### 3.5 Multivariate logistic regression analysis for patients across diverse social support categories

Single-factor analysis was performed on individuals across diverse social support categories. The findings revealed statistically significant differences (p < 0.05) in per capita disposable income of households, age, gender, disease duration, family history of DM, use of oral diabetes medication, insulin administration, and attainment of effective blood sugar control. Subsequently, employing the high-level social support and high-level utilization group as the reference, a multivariate logistic regression analysis was conducted on the four underlying social support categories among rural patients with DM to identify the influencing factors of social support classifications in diabetes. Based on the results, age, disease duration, and blood sugar control level are predictive factors for the underlying social support categories, exhibiting statistically significant differences (*p*<*0.05)* (Table 6). Patients with good blood glucose control had a higher degree of social support and were more likely to be classified into high social support groups. Additionally, patients with better social support exhibited better blood glucose control, suggesting the importance of enhancing social support for diabetic patients.

**Table 6:** Exploring the impact of different demographic factors on the latent profiles of social support among rural patients with DM.

**Item**		**C1 low-level social support utilization group**		**C2 low-level objective social support group**		**C3 moderate-level social support group**	

		**OR**	**95%CI**	**OR**	**95%CI**	**OR**	**95%CI**
Average annual per capita disposable income of households	< RMB 18,931	1.11	0.86–1.44	1.097	0.81–1.49	1.009	0.76–1.34
	≥RMB 18,931	1		1		1	
Groups based on age	18–44	7.65^***^	3.92–14.94	7.176^***^	3.47–14.86	2.006	0.91–4.42
	45–59	3.712^***^	2.73–5.04	3.823^***^	2.69–5.43	1.938^***^	1.38–2.72
	60-	1		1	.	1	
Gender	Female	0.784	0.61–1.01	0.972	0.72–1.31	0.945	0.72–1.25
	Male	1		1		1	
Groups based on disease duration	< 5	0.823	0.58–1.17	0.878	0.57–1.34	0.627^*^	0.42–0.94
	5–10	1.184	0.87–1.61	1.155	0.81–1.66	1.231	0.88–1.72
	>10	1		1		1	
Family history of DM	No	1.377	0.98–1.93	1.388	0.91–2.11	1.068	0.75–1.53
	Yes	1		1		1	1
Status of oral diabetes medication administration	No	1.268	0.97–1.66	1.249	0.91–1.71	1.134	0.84–1.53
	Yes	1		1		1	
Status of insulin administration	No	0.857	0.66–1.11	1.013	0.74–1.38	0.993	0.75–1.32
	Yes	1		1		1	
Possessing an effectively controlled blood sugar level	Yes	0.164^***^	0.12–0.22	0.176^***^	0.12–0.26	0.341^***^	0.25–0.47
	No	1		1		1	

The reference group for analysis comprised individuals with high-level social support and high-level utilization. Odds ratios (OR) were calculated with specific values assigned as follows: < RMB 18,931 = 0, RMB 18,931 or more = 1; age groups 18–44 years = 0, 45–59 years = 1, 60 years and above = 2; gender female = 0, male = 1; duration of diabetes < 5 years = 0, 5–10 years = 1, more than 10 years = 2; absence of family history = 0, presence of family history = 1; not taking oral diabetes medication = 0, taking oral diabetes medication = 1; not administering insulin = 0, administering insulin = 1; having effectively controlled blood sugar level = 0, not having effectively controlled blood sugar level = 1. ^***^p < 0.001; ^*^p < 0.05.

### 3.6 Impact of distinct social support categories on the health literacy of rural patients with DM

To further investigate the influence of distinct social support categories on the health literacy of patients, we conducted a stratified regression analysis following a single-factor examination of health literacy. This analysis involved integrating statistically significant demographic data with scores from groups categorized based on social support. The results of the multicollinearity test revealed that all explanatory variables exhibited tolerance values > 0.1 and VIF values < 5, indicating the absence of multicollinearity among factors. In the regression analysis, variables such as income, occupation, duration of the disease, family disease history, and similar factors were incorporated into the first layer, while various social support categories constituted the second layer. Subsequent to controlling for interfering variables, the findings demonstrated a notably significant impact (*p* < 0.05) of diverse social support categories on the health literacy of rural patients with DM (Table 7).

**Table 7:** Stratified regression analysis of factors affecting health literacy in rural DM patients (***n*** **=** 2,178).

	**Model 1**	**Model 2**
(Constant variable)	34.639	30.818
Average annual per capita disposable income of households	1.915^***^	1.741^***^
Employment status	0.931	0.46
Duration of the illness	−0.805^**^	−0.696^**^
Status of a familial history of DM	1.787^***^	1.285^**^
Status of oral diabetes medication administration	1.028^**^	0.754^*^
Status of insulin administration	0.719^*^	0.731^*^
Educational level	0.953^***^	0.945^***^
Number of complications	−0.328	−0.195
Possessing an effectively controlled blood sugar level or not	−8.880^***^	−6.999^***^
Low-level objective social support group	-	1.245^**^
Moderate-level social support group	-	3.719^***^
High-level social support and high-level utilization group	-	7.54^***^
*R^2^*	0.273	0.358
△*R*^2^	0.27	0.354
*F*	90.349^***^	100.567^***^

Significant difference (1) ^***^p < 0.001; (2) ^**^p < 0.01; (3) ^*^p < 0.05. Low-level social support utilization group was taken as the reference.

## 4 Discussion

### 4.1 Social support for rural patients with DM can be categorized into four distinct underlying groups

In this study, we employed LPA to explore social support categories among rural patients with DM. The low-level social support utilization group, constituting 43% of the surveyed population, exhibited the lowest aggregate score in social support. This suggests a lack of proactive initiative among rural patients with DM in seeking assistance and support. Additionally, the low-level objective social support group demonstrated slightly higher total scores compared to the low-level social support utilization group. Nevertheless, this group scored the lowest in objective social support among all groups, indicating limited direct material assistance. Together, the low-level social support utilization and low-level objective social support groups accounted for 60% of the surveyed population, highlighting a prevalent pattern of low social support among rural patients with DM, consistent with the findings of Yang et al. (17).

The remaining two patient groups constituted an equal proportion of the total surveyed population but exhibited significant differences in total scores. This discrepancy may be attributed to higher subjective social support scores in the high-level social support and high-level utilization group, suggesting better subjective emotional support for this cohort. The categorization in this study effectively delineates the social support categories and characteristics among rural patients with DM. The generally limited community support observed may be linked to delayed initiation of health services in rural China, coupled with inadequate allocation of medical resources and health education (18). Addressing the challenges in the treatment and management of rural patients with DM necessitates collaborative efforts from healthcare professionals and diverse sectors of society to enhance social support and assistance for this demographic.

### 4.2 Influential factors affecting the fundamental categories of social support among rural patients with DM

Based on the results obtained from a single-factor analysis across diverse patient groups, several factors significantly influence social support categories among rural patients with DM. These factors include per capita disposable income of households, age, gender, duration of the disease, family history of DM, use of oral diabetes medication, insulin administration, and maintenance of effectively controlled blood sugar levels.

Subsequent to this, a multivariate logistic regression analysis was carried out using the high-level social support and high-level utilization group as the reference point. The outcomes are detailed below:

1) Age and social support: individuals of a younger age are more likely to experience lower levels of social support. This observation may be attributed to the substantial work-related stress younger patients often encounter due to their pivotal roles within their families (19) and not enough attention to the disease. In addition, unlike older patients, younger individuals with DM tend to have fewer complications and milder symptoms. Family members will also neglect the care and management of patients with the extension of time. There are more people living alone among young and middle-aged patients, and they get less family support, leading to a lower receipt and utilization of social support. In clinical practice, it is imperative to place greater emphasis on both the disease and the psychological wellbeing of young rural patients diagnosed with DM. This underscores the need to counsel family members on providing adequate support to facilitate effective role transitions and maximize the utilization of social support networks during disease management.
2) Duration of disease and social support: patients with disease duration of fewer than 5 years show a higher likelihood of falling into the high-level social support and high-level utilization group. This inclination may stem from a limited understanding of the disease among patients and their families during the initial phases. Concerns about disease progression and treatment efficacy may prompt them to actively seek external support and adopt methods to regulate blood sugar levels. As DM management is a long-term chronic process, the duration of the disease corresponds to a decrease in attention from both family members and patients, provided blood sugar fluctuations do not significantly disrupt daily life. Healthcare professionals and community personnel are urged to intensify support and guidance for these patients. The course of the disease and the development of the disease should be continuously tracked, and corresponding social support should be provided in a timely manner, emphasizing the importance of disease management and increasing awareness levels.
3)Blood sugar control and social support: patients with better blood sugar control and higher levels of social support are more likely to be classified into the high-level social support and high-level utilization group. There exists a correlation between the level of blood sugar control and the extent of social support, patients with good blood glucose control level generally get a high degree of social support and have more channels to obtain disease information, which can make good use of the information obtained to manage and control blood glucose. According to the correlation analysis results in Table 4, patients with high social support also have higher health literacy level. Therefore, patients with better glycemic control have higher abilities than patients with poor glycemic control. Besides, effective social support offers patients material and spiritual assistance, including disease-related knowledge, guidance on healthy lifestyles, and emotional support (20). This support system reduces psychological stress and anxiety while aiding in the management of blood sugar levels. It is recommended that volunteers, medical personnel, and others establish social support organizations for educational and publicity campaigns in rural areas (21). Additionally, hospitals should provide regular medical services, resource support at the grassroots level, establish DM clinics, and enhance the promotion of DM-related knowledge in rural areas (22).

### 4.3 Variations in health literacy among rural patients with DM across diverse social support categories and the associated correlation between these support classifications and health literacy levels

In this study, we conducted inter-group comparisons of health literacy scores and scores across various dimensions among different patient groups. These groups received varying levels of underlying social support. The results indicated a positive correlation between social support scores and both the overall health literacy score and scores across all dimensions. Specifically, higher social support scores were associated with higher health literacy scores. This correlation analysis was further explored, leading to the conclusion that health literacy among rural patients with DM is positively correlated with the level of social support received.

Consistent with the findings of Souza et al., (23) our results suggest that increasing social support can positively impact the health literacy of rural patients with DM. Patients with a high degree of social support have a high level of support from family, friends and colleagues, and have a strong ability to obtain information. They can well handle, distinguish and apply health information of diseases, and better manage their blood sugar. Single-factor analysis demonstrated statistically significant differences in health literacy levels among rural patients with DM based on the categories of social support they received (*p* < 0.05). To control for interference from demographic variables, we conducted stratified regression analysis, revealing significant differences in all dimensions of health literacy between patient groups with distinct social support categories.

Our findings underscore the importance of enhancing social support for rural patients with DM, as it can significantly benefit their health literacy across all dimensions. To improve health literacy and blood sugar management capabilities in this population, efforts should be directed toward increasing investment in social support resources for patients with DM in rural areas. Strengthening the construction of grassroots healthcare teams and implementing peer support interventions (24) and family support interventions (25) within rural communities are recommended. Alternatively, developing a user-friendly remote platform for managing DM data (26) can be considered. These measures aim to address the information needs of patients, enhance their subjective initiative, and improve the utilization of social support, ultimately elevating the level of health literacy.

The limitation of this study should also be addressed. Firstly, although the results suggest that patients with good blood glucose control had a higher degree of social support and were more likely to be classified into high social support groups, the cross-sectional nature of the data means that temporality cannot be established. Therefore, the directionality should be interpreted with caution. Secondly, excluding patients in the acute phase of the disease due to ethical considerations limits this study, as this group may be among those most in need of social support.

## 5 Conclusion

In conclusion, the constrained accessibility of social support resources in rural areas significantly affects the health literacy and blood sugar management capabilities of individuals with DM. Healthcare practitioners should enhance initiatives to provide social support to rural patients with DM, with particular emphasis on optimizing social support utilization and delivering objective assistance. Addressing the social support hurdles encountered by rural patients with DM requires a coordinated approach involving various stakeholders. It is imperative to exert concerted efforts in delivering medical resources, health education, economic support, social connections, and other relevant interventions to empower these patients in effectively managing their blood sugar levels and elevating their overall quality of life.

## Data Availability

The original contributions presented in the study are included in the article/supplementary material, further inquiries can be directed to the corresponding author.
